# Theoretical Study of Hydrogen on LaFeO_3_ (010) Surface Adsorption and Subsurface Diffusion

**DOI:** 10.3390/ma11122347

**Published:** 2018-11-22

**Authors:** Changchang Pan, Yuhong Chen, Meiling Zhang, Lihua Yuan, Cairong Zhang

**Affiliations:** 1 School of Science, Lanzhou University of Technology, Lanzhou 730050, China; ccpanzw@163.com (C.P.); zhangml_2000@126.com (M.Z.); yuanlh@lut.cn (L.Y.); zhcrxy@lut.cn (C.Z.); 2 State Key Laboratory of Advanced Processing and Recycling of Non-Ferrous Metals, Lanzhou University of Technology, Lanzhou 730050, China; 3 CAS Key Laboratory of Design and Assembly of Functional Nanostructures, and Fujian Provincial Key Laboratory of Nanomaterials, Fujian Institute of Research on the Structure of Matter, Chinese Academy of Sciences, Fuzhou 350002, China

**Keywords:** density functional theory, LaFeO_3_, surface adsorption, subsurface occupy, transition state

## Abstract

Based on density functional theory, this paper studies the adsorption and the subsurface occupation by H on LaFeO_3_ (010) surface and their corresponding transition states. As shown from the results, the best storage positions of hydrogen are on the O top position of the LaFeO_3_ (010) surface and the interstice near the oxygen of the subsurface. In addition, the position of surface Fe atom can also store hydrogen, but H atom prefers to adsorb on O atom first. Whether the H atom is adsorbed on O or Fe atom, it is easy diffuse to the nearby more stable O atom. However, the diffusion between the Fe atoms is difficult to occur. The main diffusion path of the H atom from the surface to the subsurface is the process of inward layer by layer around the O atom. With the fracture of the old H–O bond and the formation of the new H–O bond, the H is around O atom to constantly repeat the process of a hopping-rotational diffusion. H diffuses through the nearest neighbor position, which is more favorable than the direct diffusion.

## 1. Introduction

As a kind of electrode material, ABO_3_ perovskite oxide not only possesses good electrochemical activity and high discharge capacity, but also has a certain universality for the electrochemical hydrogen storage phenomenon [1]. Therefore, the use of ABO_3_ oxide as a new anode material for Ni MH battery is not only feasible, but also has such advantages as low cost, easy activation, high discharge capacity and good chemical stability, which are of great value [2]. The study of hydrogen storage mechanism of ABO_3_ oxides mainly focused on high-temperature proton conductors at the preliminary stage [3]. Because ABO_3_ oxides produce oxygen vacancies after being doped with rare earth ions, the water vapor (or hydrogen) in the external environment of oxides react with oxygen vacancies (or electron holes) to generate protons [4]. This is a defect reaction and the reaction equations are

(1) $$\;H_{2}O+V_{O^{\mathrm{OO}}}=2H^{+}+O_{O^{X}}\;$$

(2) $$\;H_{2}+2h=2H^{+}\;$$

where $V_{O^{\mathrm{OO}}}$ refers to the oxygen vacancy, $O_{O^{X}}$ refers to oxygen ion on normal lattice, h refers to electron hole, and $H^{+}$ refers to interstitial proton. The conduction mechanisms of protons in LaFeO_3_ oxides have been reported [5], it is generally believed that a weak O–H bond is formed between the proton adsorbed in oxides and the oxygen ion. Under an external electric field, the old O–H bonds break. During the proton precession, a new weak O–H bond is formed between the proton and the adjacent oxygen ion, the H is around O atom to constantly repeat the process of a hopping-rotational diffusion. The proton repeats the process of hopping, resulting in rotational conduction [6]. Mandal [7] et al. found that at atmospheric pressure, H_2_ containing environment and 180~260 °C, the Pt coated on AMnO_3_ (A = Ca, Sr, Ba) oxide surfaces could achieve reversible hydrogen storage capacity up to 1.25 wt%, and the chemical formula was AMnO_3_H_3_. Thinking of hydrogen as a proton entering the oxide, forming covalent bonds with Mn and reducing Mn^4+^ to Mn^3+^, thus the hydrogen forms OH^−^ with the oxygen on the octahedral plane. Esaka [8] argues that the perovskite type oxides ACe_1−x_M_x_O_3−δ_ (A = Sr, Ba; M = rare earth element) readily adsorb protons and reduce Ce^4+^ to Ce^3+^. Apart from protons, hydrogen atoms can also be absorbed by perovskite materials. Following the electrochemical hydrogen storage via mass spectrometry, X ray photoelectron spectroscopy and other methods, Chen Yungui et al. [9] analyzed the mechanism of hydrogen storage with LaFeO_3_ and the valence change of Fe elements. They considered that hydrogen was dissolved and stored in the LaFeO_3_ lattice in the atomic state instead of the proton, and the semi saturated d orbitals of Fe^3+^ form covalent bonds with H in the process of hydrogen storage, leading to the partial reduction from Fe^3+^ states to Fe^2+^. The above studies show that, regardless of whether hydrogens are present as protons or in atomic states in the ABO_3_ lattice, the B terminal elements in the ABO_3_ structure will change their valence state when the hydrogen is stored.

In addition, as concluded from on literature analysis [10,11,12], the exchange current density and H atom diffusion coefficient of La_1−x_Sr_x_MO_3_ (M = Fe, Cr) electrodes are much smaller than those of hydrogen storage alloy electrodes, which means that the surface catalytic activity of the electrode and the diffusion of hydrogen atoms in the La_1−x_Sr_x_MO_3_ bulks are important factors affecting electrochemical performances. However, theoretical and experimental studies on how hydrogen atom diffusions of ABO_3_ type oxides affect their hydrogen storage properties have not yet been reported. At present, research on the hydrogen storage properties of ABO_3_ type oxides is focused on electrochemical properties [13,14]. The mechanism of hydrogen storage in gas–solid state is not clear, such as the occupation of H in material crystals and the diffusion of H in the bulk; the mechanism of the hydrogen storage reaction of ABO_3_ type oxide materials is also unknown, and there are still no reasonable theoretical explanations for some important experimental observations. As mentioned in References [7,8,9,10,11,12], (1) the relationship between the hydrogen storage capacity of LaFeO_3_ and its maximum discharge capacity (625 mAh/g) in electrochemical reactions does not match. (2) The electrochemical performance of La_1−x_Sr_x_MO_3_ (M = Fe, Cr; x = 0–0.4) is closely related to the ambient temperature, especially the discharge capacity, for example: when the temperature is higher than 40 °C, the discharge capacity of the electrode will increase significantly, but the reason is not clear. Therefore, this paper analyzes the occupation positions of the H in the subsurface by analyzing the adsorption of the H atom on surfaces and the occupation positions of the H in the subsurface. In addition, based on the transition state theory, the possible diffusion pathways are determined by searching the transition states of H on material surfaces and subsurface and comparing the energy barriers of diffusion paths of H atom.

## 2. Calculation Method and Model

**Figure 1:**
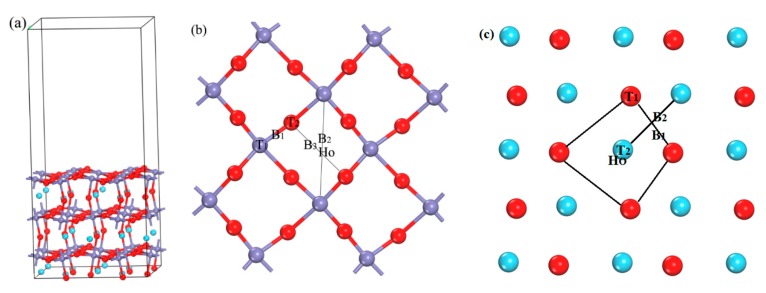
The structural schematic of LaFeO_3_ (010) surface (**a**), the initial structure of H atoms adsorbed on the FeO_2_-terminated surface (**b**) and the initial structure of H atoms adsorbed on the LaO-terminated surface (**c**) (T stands for the top position, B represents the bridge position, while Ho represents the hollow position). lanthanum in cyan, iron in light blue and oxygen in red.

The first principles calculations were performed using the Cambridge Sequential Total Energy Package (CASTEP 7.1) computer code [15] in the framework of DFT, and the DFT evaluation was based on the plane-wave expansion. The generalized gradient approximation (GGA) in the form of Perdew-Burke-Ernzerhof exchange-correlation functional [16], and the ultrasoft pseudopotential [17] is adopted to describe the electron-ion interaction. We treated the O (2s, 2p), Fe (3s, 3p, 3d, 4s), and La (5s, 5p, 5d, 6s) electrons as valence states, whereas the remaining electrons were kept frozen as core states. The partial occupancies were calculated using finite temperature approaches—smearing methods [18]—and the smearing was 0.1 eV. The DFT + U approach was used to describe the localized 3d electronic states in Fe. In line with earlier work [19,20], we choose a Hubbard-U value of 4.0 eV for the Fe 3d states. Spin-polarized calculations have been applied throughout. The LaFeO_3_ crystal is an orthorhombic perovskite structure with space group of Pnma (No. 62) and 20 atoms in each cell. It is found through previous calculations that the (010) surface is the most stable surface [21]. The adsorption energies of H on the third layers of the four, six and eight surface structures were tested. It is found that the adsorption energy difference of H in the third layers of the six and eight surface structures is similar. Moreover, because the calculation system contains La atoms, the calculation involves f electrons, and the computation is very large, so we chose six layers of surface structure. The surface (26 atoms) of the 2 × 2 supercell is large enough to eliminate the influence of the image molecule, so in order to reduce the amount of computation, we select the supercell of 2 × 2. Therefore, comprehensively considering the accuracy and efficiency of the calculation, a supercell (2 × 2) of 6 layer atoms is constructed to simulate the calculations, as shown in Figure 1a, which contains 120 atoms. The necessary convergence tests [22] are performed for the cut-off energy and the k-point mesh in the Brillouin zone, and the corresponding test charts as shown in Figure S1. The kinetic energy converged to 1 meV/atom, the cut-off energy is set to 700 eV, and the k-point mesh in the Brillouin zone is 7 × 7 × 1; self consistent field convergence is 2 × 10^−6^ eV, the force on each atom is less than 0.02 eV/Å, and the vacuum region is 15 Å to ensure that the vacuum thickness is large enough to avoid spurious interactions between the slabs. Because the main research of this paper is chemical adsorption, not molecular adsorption, it is the adsorption process of H atom, and H–O is the covalent bond. However, similar systems also do not consider vdW in exploring hydrogen storage process [5,23]. These parameters are used to test the structure of LaFeO_3_ crystal: The optimized lattice parameters are a = 5.4930 Å, b = 7.7558 Å and c = 5.5111 Å respectively, complying well which is in good agreement with the experimental results 5.5595, 7.8498 and 5.5509 Å [24], which shows that the theoretical calculation accuracy is good and the calculation model is reliable.

For the transition state calculations, the H atom is placed at the top position (T), bridge position (B), and hollow position (Ho) of the surface, as shown in the Figure 1b, as well as the symmetrical position of the near and interstice of the subsurface. After optimization, the adsorption positions of H atom on the LaFeO_3_ (010) surface and the occupied position of the subsurface are obtained. Then the most stable adsorption position of the surface is taken as the initial structure, the best-occupied position of the subsurface is taken as the final structure, and the transition states [25,26,27] are searched by combining linear synchronous transit (LST) with quadratic synchronous transit (QST) methods [28]. We used the functions of TS-Confirmation [29] to confirm the transition state. For detailed instructions, see supplementary material.

The adsorption energies [30] of H atoms are:

(3) $$\;E_{\mathrm{ads}-H}=\frac{E_{{\mathrm{LaFeO}}_{3}\left(010\right)/H}-E_{{\mathrm{LaFeO}}_{3}\left(010\right)}-{\mathrm{NE}}_{H}}{N}\;$$

*N* is the total number of H atoms, $E_{{\mathrm{LaFeO}}_{3}\left(010\right)}$ is the total energy of the surface of LaFeO_3_ (010), E_H_ is the energy of a single H atom, and $E_{{\mathrm{LaFeO}}_{3}\left(010\right)/H}$ is the total energy due to the adsorption or occupation of H. Based on this definition, the value is negative, indicating stable structure. Furthermore, the higher the absolute value, the more stable the structure.

## 3. Results and Discussion

### 3.1. Adsorption of H Atom on the Surface and Occupation in the Subsurface

#### 3.1.1. Adsorption of H Atom on Surface

**Figure 2:**
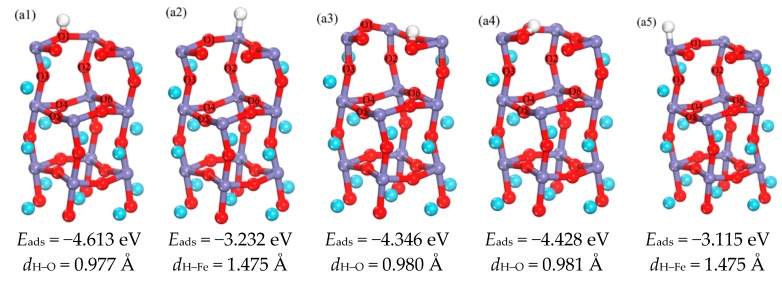
(**a1**–**a5**) are the schematic diagram of the optimized structure of H adsorbed on the surface and the corresponding adsorption energy and bond length (in order to make the image more concise and clear, choose to hide some atomic regions).

The LaFeO_3_ (010) surface has two kinds of possible terminations, i.e., FeO_2_-terminated and LaO-terminated surfaces respectively, and the initial adsorption positions include top position (T), bridge position (B) and hollow position (Ho), as shown in the Figure 1b,c. It is found that the adsorption of H atom on the LaO-terminated surface is not favorable. In addition to the O atoms placed on the top of La position escaping from the surface, the other adsorption sites H and O atoms combined and escaped from the surface together. At the same time, it has been found that LaO-terminated surfaces are difficult to be formed [31]. Therefore, only the FeO_2_-terminated surface is discussed in this paper. Figure 2(a1–a5) shows the optimized structures of H atoms in the surface. It can be seen that the optimized H–O bond lengths are in the range of 0.977–0.981 Å, This is similar to the H–O bond length 0.978 Å of H_2_O molecules calculated by Lie [32], and so the strong H–O bond is formed. In addition, the H–Fe bond length is 1.475 Å. The bond angles of ∠HOFe for a1–a3 are 99.7°, 98.3°, and 68.5°, meanwhile the bond angles of ∠HFeO for a2 and a5 are 93.6°and 94.5°, respectively. Therefore, the orientation of the O–H bond is different, and different structures are obtained. It can be concluded from the comparison of adsorption energies, that the best adsorption position of H atom in the surface layer is on top of the O1 atom (a1), and that the Fe position can also store hydrogen [21].

#### 3.1.2. The Occupation of H in the Subsurface

**Figure 3:**
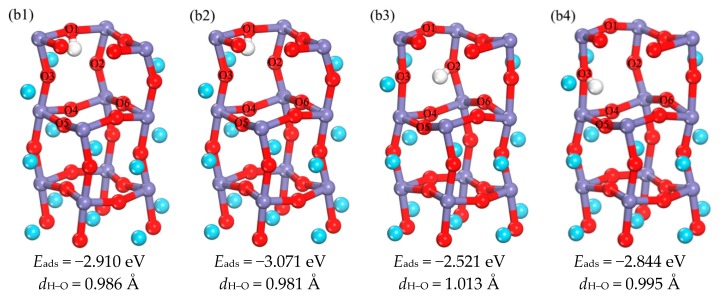
(**b1**–**b4**) are the schematic diagram of the optimized structure of H occupied in the sub-surface, and the corresponding adsorption energy and bond length (in order to make the image more concise and clear, choose to hide some atomic regions).

There are mainly four initial positions for the H in the sub-surface: the position near the Fe or O atom (Fe site or O site), bridge position (B), center position of triangle (Fc), and the center of a tetrahedron (Te), as shown in the Figure S2. Figure 3(b1–b4) indicates the optimized structures of H in the sub-surface layer, and the optimized H–O bond lengths are in the range of 0.981–1.013 Å. The results in b1 and b2 show differences in the orientation of O–H bonds. They occupy the position below the O1 atom located in the interstice between the surface and sub-surface layer, and the difference between the two adsorption energies is 0.161 eV; so b1 and b2 are both regarded as possible intermediates. The adsorption energies of b3 and b4 show that the adsorption energy of b4 is smaller and the structure is more stable; therefore, b4 is the most favorable occupation position in the sub-surface layer, that is to say, the best occupation position of H in the sub-surface layer is near the O3 atoms.

**Figure 4:**
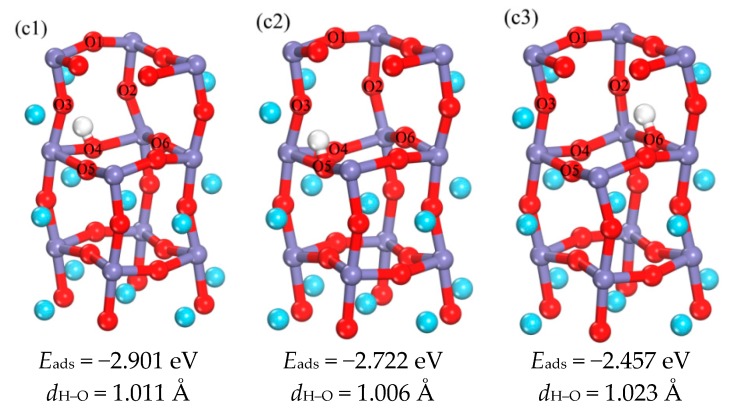
(**c1**–**c3**) are the schematic diagram of the optimized structure of H occupied in the third layer, and the corresponding adsorption energy and bond length (in order to make the image more concise and clear, choose to hide some atomic regions).

Figure S3 and Figure 4(c1–c3) provide the initial occupying positions and the optimized structures of H in the third layer, respectively. The optimized H–O bond lengths are in the range of 1.006–1.023 Å. According to the adsorption energies of c1–c3 structures, the adsorption energy of c1 is the more negative, which means this is the best occupation position. However, it is found from the schematic diagram of b4 which is the best occupation position in the sub-surface layer, that H prefer to O5. So it is speculated that c2 may be the best occupation position for the third layer. In summary, both c1 and c2 are considered as the most favorable occupation positions in the third phase, i.e., the best occupation positions in the third layer are near the O4 atom or the O5 atom.

**Figure 5:**
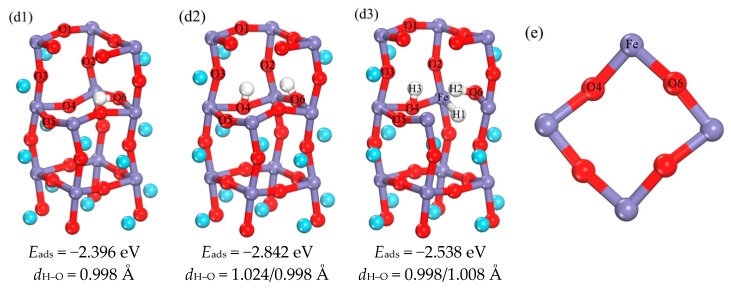
(**d1**–**d3**) are the schematic diagram of optimized structure of different number of H in the third layer, and the corresponding adsorption energy and bond length. (**e**) Is a third layer overlook map (in order to make the image more concise and clear, choose to hide some atomic regions).

The surface layer Fe atom can store hydrogen and Fe atoms also occur in the third layer, but H do not occupy any positions near the Fe atom in all the optimized structures. Therefore, a series of tests was carried out to detect whether the H could be stored near the Fe atom in the third layer. As shown in Figure 5(d1–d3), a series of structures was optimized by placing different amounts of H in the third layer. Figure 5e is a top view of the third phase, when one H atom is placed in the center of triangle (O4, O6 and Fe atoms), it can be seen from d1 structure, that the H atom occupies the interstice near the O6 atom. When one H atom is placed in the center of triangle and another H atom is placed near to the O6 atom to eliminate the interference of the O6 atom, it is found that the two H atoms occupy sites near the O4 and O6 atoms, respectively. On the basis of the d2 structure, one more H atom is placed near the Fe atom to eliminate the interference of the O4 atom. After optimizing the structure, such as d3, the three H atoms occupy sites near the O4, O6 and Fe atoms, respectively. Therefore, it is not only proved that the Fe^3+^ ions can store hydrogen in the third layer, the adsorption of H in bulk Fe^3+^ ions was also investigated in literature [33]. It is found that the most stable absorption configuration corresponds to the position of interstitial tetrahedron, which is consistent with the result that the position of Fe can store hydrogen. However H atoms are easier to be stored on O atoms. If hydrogen is saturated near oxygen, the excess H will be stored in the interstitial sites near Fe atoms.

#### 3.1.3. Analysis of the Properties of d3 Structure

**Figure 6:**
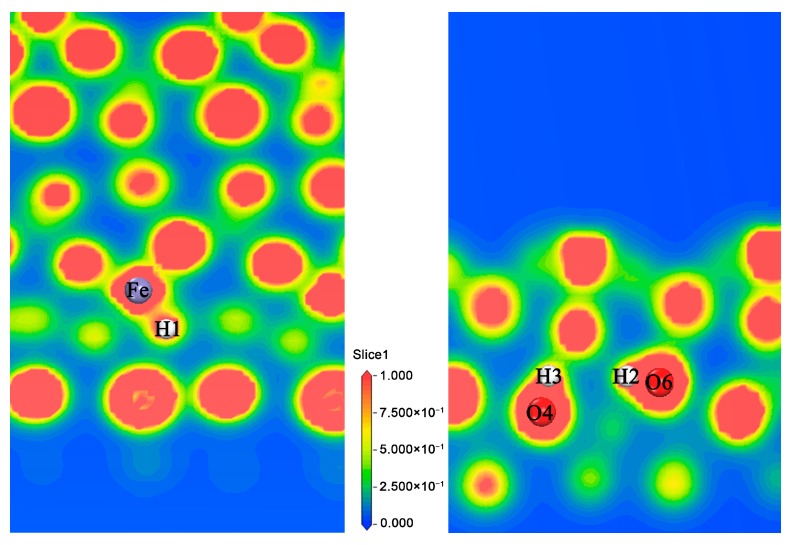
Electron localization function of the d3 structure.

The d3 structure proves that the Fe atom can store hydrogen in the third layer, so the hydrogen storage performance of the Fe site is explored. The Mulliken analysis [34,35,36,37] and the electron localization function (ELF) [38,39] are calculated. We calculated the structures of pure host (Figure 1a), adsorbed 2 H (Figure 5(d2)) and adsorbed 3 H (Figure 5(d3)) respectively, and analyzed the corresponding charge population, as shown in Table 1. Compared with the pure host structure and the adsorption of 3 H, the valence of Fe decreases, and 0.14 electrons was obtained, the valence of H bonded with Fe decreases, and 0.33 electrons was obtained, the valence of O4 and O6 decreases, 0.01 and 0.02 electrons were obtained respectively, while the valence of H2 and H3 increases, and 0.23 electrons were lost. Compared the adsorption of 2 H and the adsorption of 3 H, the conclusion is similar. The valence of Fe decreases, and 0.18 electrons was obtained, the valence of H bonded with Fe decreases, and 0.33 electrons was obtained, the valence of O4 and O6 decreases, 0.03 and 0.04 electrons were obtained respectively, while the valence of H2 and H3 increases, 0.03 and 0.02 electrons were lost respectively. Therefore, the calculated results are in agreement with the experimental results [9]. To further analyze the bonding characteristics among three different structures, Table 2 lists the bond populations and bond lengths for the various elements. The bond charge population of O–H is larger than the Fe–H, so the O–H bond is stronger than the Fe–H bond. The electron localization function (ELF) is also a tool for discussing charge transfer. In Figure 6, the electron density distributions of Fe–H and O–H on d3 are shown; here, highly localized electrons show the strongest covalent bond on ELF = 1, (red parts), a metallic bond on ELF = 0.5 and stronger ionic bonding on 0 ≤ ELF < 0.5 [40]. As shown, there is an obvious electron density overlap between Fe and H, as well as between O and H, and Fe–H in the yellow region (ELF < 0.5), while O–H is in the red area (ELF = 1), therefore, Fe–H is approaching ionic bond, and the O–H bond is a typical covalent bond.

### 3.2. Diffusion of H on the Surface and in the Subsurface

#### 3.2.1. Diffusion of H Atom on the Surface

**Figure 7:**
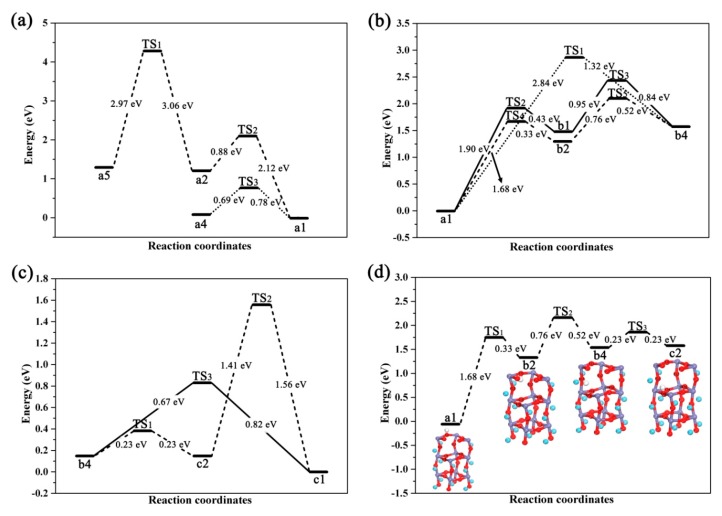
Diffusion energy barrier of H in surface and subsurface (Ordinate is relative energy, (**a**,**b**,**d**) are relative a1 state, and (**c**) is relative c1 state).

In the process of H atom being adsorbed on the surfaces, the top position of O1 atom is the most favorable position, but a Fe atom can also adsorb H; Therefore, when H is adsorbed on O or Fe atom, the diffusion of H atom between O–O, O–Fe and Fe–Fe are calculated respectively. The corresponding diffusion energy barriers are shown in Figure 7a, which indicate the diffusion barriers for the forward and reverse paths of a2 (Figure 2(a2)) to a1 (Figure 2(a1)) are 0.88 eV and 2.12 eV, respectively. It is known that the adsorbed H atom on the surface Fe diffuses easily compared to the diffusion on the neighboring O atom; the diffusion barriers for the forward and reverse paths of a4 to a1 are 0.69 eV and 0.78 eV, respectively. The adsorption energy of a1 is more negative and the structure is more stable, so H readily diffuses from O to neighboring more stable O positions [41]. The diffusion barriers of the forward and reverse paths for a5 to a2 are 2.97 eV and 3.06 eV, respectively, and it can be seen that the diffusion between Fe atoms is much less favorable. Therefore, when a large number of H atoms are adsorbed on the surface, H will first be adsorbed on the O atoms, and subsequently diffuses to neighboring more stable O sites. In this process, some of the H atoms are adsorbed on the Fe atoms, but the H adsorbed on the Fe atoms diffuse easily to the neighboring O atoms. When hydrogen is adsorbed by surface oxygen atoms, the excess H will be adsorbed on the Fe atoms, while the H atoms hardly diffuse between Fe sites.

#### 3.2.2. Diffusion of H from Surface to Sub-Surface Layer

From the study of the adsorption of H on the surface and the occupation in the sub-surface layer, we know that a1 is the most favorable adsorption position, b4 is the best occupation position of the sub-surface layer, and b1 and b2 are the intermediates concerning the surface to sub-surface layer diffusion. Therefore, the three diffusion paths of the two-step diffusion a1→b1→b4 and a1→b2→b4, and the direct diffusion of a1→b4 are considered. As shown in Figure 7b, the energy barriers for the forward and reverse diffusion paths of a1→b1→b4 are 2.85 eV and 1.27 eV, respectively, and the energy barriers for the forward and reverse diffusion paths of a1→b2→b4 are 2.44 eV and 0.85 eV, respectively. Comparing these two step paths, we can see that when b2 is used as an intermediate, both the forward diffusion and the reverse diffusion paths have a lower energy barrier, so the substep diffusion is more favorable to the diffusion path of a1→b2→b4. The energy barrier of the forward and reverse diffusion paths for the direct diffusion of a1→b4 are 2.84 eV and 1.32 eV, respectively, and the required barriers are higher than for a1→b2→b4. Therefore, a1→b2→b4 is the most favorable diffusion path for surface to sub-surface layer diffusion, and H diffuses from the surface to sub-surface layer, through the nearest neighbor position, which is more favorable than the direct diffusion. However, this conclusion is similar to the result in literature [30], that is, the diffusion path of H atom on the surface of Ni_3_Fe (111) is similar.

#### 3.2.3. The Diffusion of H from the Sub-Surface Layer to the Third Layer

The most favorable position of H in the sub-surface layer is b4. While c1 and c2 are probably the most favorable occupation positions for the third layer. We consider the two diffusion paths of b4→c1 and b4→c2. As shown in Figure 7c, the energy barriers for the forward and reverse diffusion paths of b4→c1 are 0.67 eV and 0.82 eV, respectively. The energy barrier for the forward and reverse diffusion paths of b4→c2 are 0.23 eV. By comparison, it is known that the diffusion barrier for the diffusion path of b4→c2 is lower, while compared with c1 and c2, the adsorption energy of c1 is smaller and the resulting structure is more stable. Therefore, it is speculated that the H will diffuse from b4 to c2 and then diffuse from c2 to c1, so the forward and reverse diffusion energy barriers of c2→c1 are calculated to be 1.41 eV and 1.56 eV, respectively. It is found that the barrier value of the diffusion process is higher. Therefore, c2 is the best occupation position for the third layer, and b4→c2 is the most favorable diffusion path for the sub-surface layer to the third layer.

#### 3.2.4. The Main Diffusion Path of H Diffusion from the Surface to the Subsurface

In summary, H initially is adsorbed at the most stable position of the surface. Subsequently, H enters the sub-surface layer to occupy a site close to the intermediate (as shown in b2 in Figure 3, H storage in the interstitial site between the surface and sub-surface), then passes through the intermediate to the sub-surface layer, and finally spreads to the third layer. Overall, the main diffusion path is a1→b2→b4→c2 (Figure 7d). And as seen from the diffusion energy barrier diagram of Figure 7d, the forward diffusion energy barriers are 1.68 eV, 0.76 eV and 0.23 eV, respectively. Therefore, if the barrier of 1.68 eV is crossed, H can diffuse from surface to the sub-surface layer, and once H enters the sub-surface layer, it will diffuse more easily downward. These results are similar to the conclusions in References [30,42]. The energy barriers for reverse diffusion are 0.23 eV, 0.52 eV and 0.33 eV, respectively. It can be seen that the required barrier for reverse diffusion is lower. Furthermore, the structure of the final state c2 possesses higher energy than the initial state a1. The diffusion path of a1→b2→b4→c2 is essentially the process of storing H from the surface to the interior, while the diffusion path of c2→b4→b2→a1 is the process of releasing H from the interior to the surface, that is to say, the hydrogen release process is more likely to occur than the hydrogen storage process. By analyzing the schematic diagram of structure of the main diffusion path after optimization, we found that the process of diffusion of H is revolving around the O atom and inward layer by layer [6,43].

## 4. Summary

Based on density functional theory, we calculated the adsorption of H atoms on LaFeO_3_ (010) surface and the incorporation process in the subsurface and their corresponding diffusion process. The conclusions are as follows:

(1) The best adsorption position of H atom is on the top of the surface O1 atom position, and the Fe atom position can also store hydrogen. The best occupation position of the H atom in the sub-surface layer is near the O3 atom, and the best occupation position of the H atom in the third layer is near the O5 atom. It is also found that the Fe atom can store hydrogen in the third layer, but that H is more favorably stored on O sites.

(2) During the diffusion among surface atom, H adsorbed on surface Fe easily diffuses to the neighboring O site, and the H atom adsorbed on O atom can also diffuse to neighboring more stable O atom, but the diffusion between the Fe atoms is less probable to occur.

(3) H diffuses from the surface to the sub-surface layer through the nearest neighbor position, which is more favorable than the direct diffusion. The main diffusion path of the H from the surface to the subsurface is a1→b2→b4→c2. The diffusion process of H is revolving around the O atom and inward layer by layer. When H atoms diffuse inward, with the fracture of the old H–O bond and the formation of the new H–O bond, the H is around O atom to constantly repeat the process of a hopping-rotational diffusion.

In this paper, we investigate that Fe in the subsurface can also store hydrogen, the hydrogen storage process is reversible, and the hydrogen release process is more likely to occur than the hydrogen storage process, which is compared to the storage process. The hydrogen storage and release process is not spontaneous and requires some external adjustments, such as under an external electric field or temperature and hydrogen pressure. Through the analysis of the properties of Fe in the third layer, we find that Fe loses electrons in the hydrogen storage process, which is agreement with the experimental results, Fe get electron, leading to the partial reduction from Fe^3+^ states to Fe^2+^.

## Figures and Tables

**Table 1:** The charge population of the three structures of pure host (Figure 1a), adsorbed 2 H (Figure 5(d2)) and adsorbed 3 H (Figure 5(d3)).

Atom	Pure Host (e)				Adsorption 2 H (e)				Adsorption 3 H (e)			
	s	p	d	Charge	s	p	d	Charge	s	p	d	Charge
Fe	0.33	0.58	6.51	0.59	0.32	0.45	6.60	0.63	0.33	0.57	6.65	0.45
O4	1.86	4.86	-	−0.72	1.83	4.91	-	−0.74	1.83	4.88	-	−0.71
O6	1.85	4.86	-	−0.71	1.82	4.91	-	−0.73	1.81	4.87	-	−0.69
H1	1.00	-	-	-	1.00	-	-	-	1.33	-	-	−0.33
H2	1.00	-	-	-	0.80	-	-	0.20	0.77	-	-	0.23
H3	1.00	-	-	-	0.79	-	-	0.21	0.77	-	-	0.23

**Table 2:** The bond population of the three structures of pure host (Figure 1a), adsorbed 2 H (Figure 5(d2)) and adsorbed 3 H (Figure 5(d3)).

Bond	Population (e)			Length (Å)		
	Pure Host	Adsorption 2 H	Adsorption 3 H	Pure Host	Adsorption 2 H	Adsorption 3 H
Fe–H1	-	-	0.50	-	-	1.534
O4–H3	-	0.70	0.70	-	0.998	1.005
O6–H2	-	0.68	0.72	-	1.024	0.995
Fe–O4	0.33	0.12	0.15	1.984	2.126	2.125
Fe–O6	0.30	0.11	−0.23	1.974	2.087	2.807

## Supplementary Materials

The following are available online at http://www.mdpi.com/1996-1944/11/12/2347/s1, Figure S1: Test convergence diagram of cut-off energy, k-point mesh and vacuum region, Figure S2: There are different initial positions for the H in the sub-surface. Square indicates initial placement, Figure S3: There are different initial positions for the H in the third layer. Square indicates initial placement.
