# Supplementary material for: Theoretical Study of Hydrogen on LaFeO3 (010) Surface Adsorption and Subsurface Diffusion
Source: Materials (Basel). 2018 Nov 22;11(12):2347. doi: 10.3390/ma11122347 (PMC6316970; doi:10.3390/ma11122347)
Supplement: Supplementary file 1 [file materials-11-02347-s001.pdf]

Supplementary Materials

Theoretical Study of Hydrogen on $LaFeO_3$ (010) Surface Adsorption and Subsurface Diffusion

Changchang Pan [1,2,3], Yuhong Chen [1,2,*], Meiling Zhang [1], Lihua Yuan [1] and Cairong Zhang [1,2]

1 School of Science, Lanzhou University of Technology, Lanzhou 730050, China; ccpanzw@163.com (C.P.); zhangml_2000@126.com (M.Z.); yuanlh@lut.cn (L.Y.); zhcrxy@lut.cn (C.Z.)
2 State Key Laboratory of Advanced Processing and Recycling of Non-ferrous Metals, Lanzhou University of Technology, Lanzhou 730050, China
3 CAS Key Laboratory of Design and Assembly of Functional Nanostructures, and Fujian Provincial Key Laboratory of Nanomaterials, Fujian Institute of Research on the Structure of Matter, Chinese Academy of Sciences, Fuzhou 350002, China
* Correspondence: chenyh@lut.cn; Tel.: +86-931-297-3780

Calculation Method and Model

The functions of TS-Confirmation are to confirm the transition state by Nudged Elastic Band method. After TS Search get the track file (a .xtd file), TS-Confirmation uses NEB method to optimize the reasonable reaction path on the basis of the trace file, and obtains the energy of each point on the path. By analyzing the extreme energy points on the reaction path, the maximum energy is at transition state and the minimum energy is at metastable state. After TS Search is finished, TS-Confirmation is used to analyze whether the energy curve is reasonable or not and whether the transition states are missing. Because the system is large and the frequency calculation is too time-consuming, so the transition state is confirm by TS Confirmation.

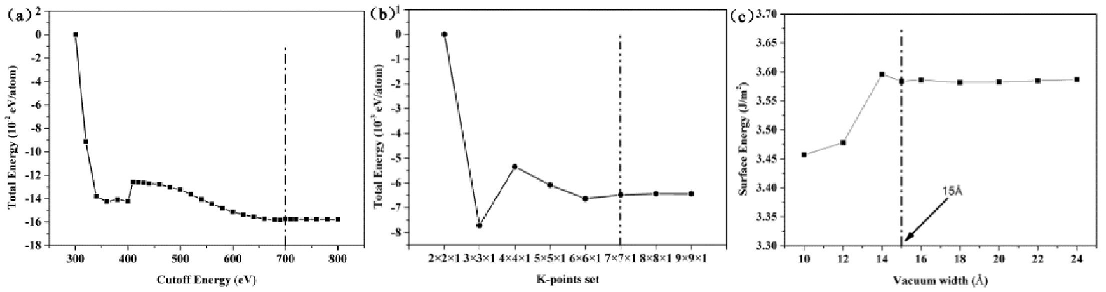

Figure S1 Test convergence diagram of cut-off energy, k-point mesh and vacuum region.

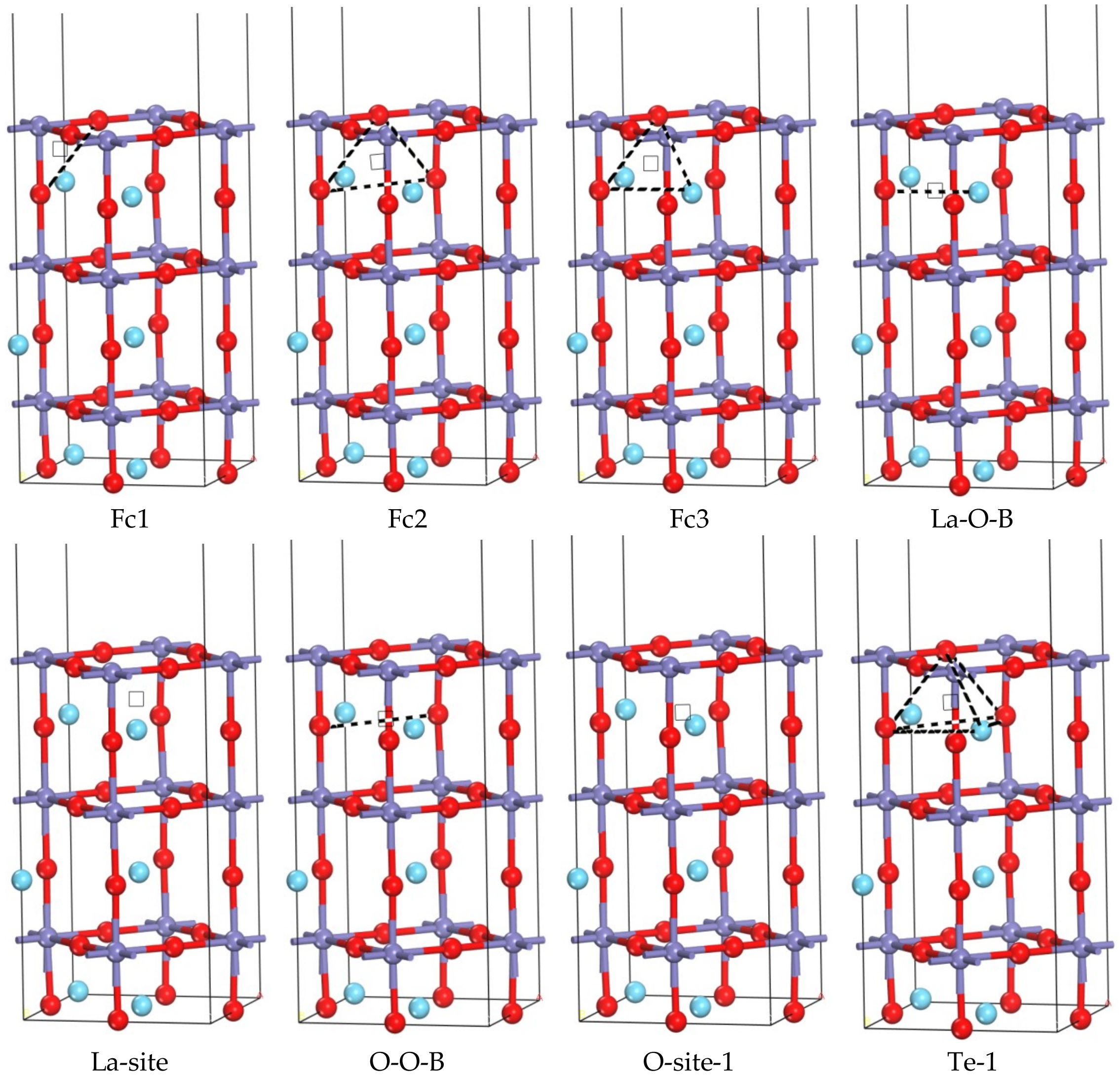

Figure S2 There are different initial positions for the H in the sub-surface. Square indicates initial placement.

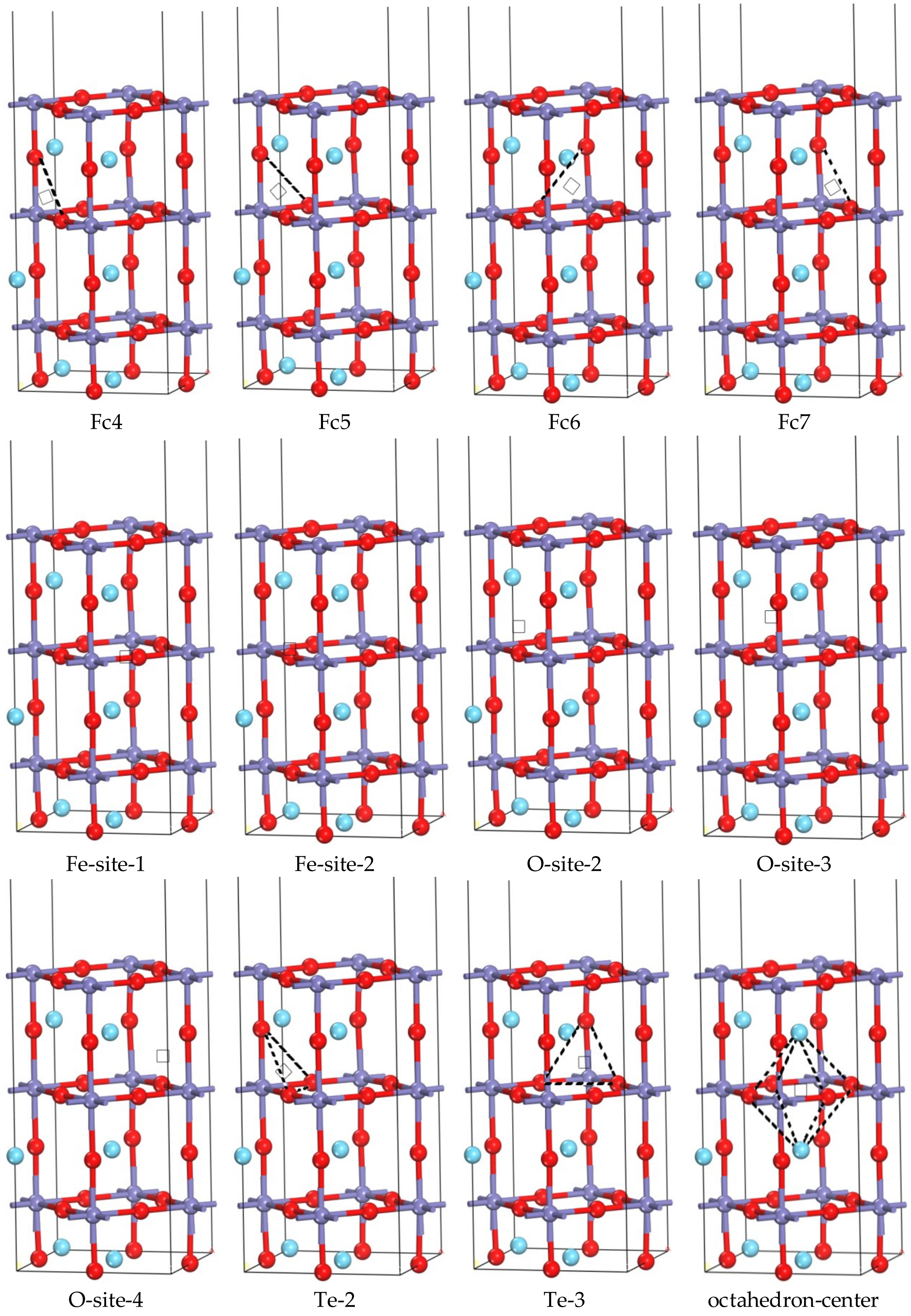

Figure S3 There are different initial positions for the H in the third layer. Square indicates initial placement.
